# Retraction: Lycium barbarum polysaccharides inhibit proliferation and migration of bladder cancer cell lines BIU87 by suppressing Pi3K/AKT pathway

**DOI:** 10.18632/oncotarget.28839

**Published:** 2026-04-24

**Authors:** Xian-Jun Zhang, Hong-Yuan Yu, Yong-Jian Cai, Mang Ke

**Affiliations:** ^1^Department of Urology, Taizhou Hospital of Zhejiang Province, Wenzhou Medical University, Linhai, Zhejiang Province, PR China

**This article has been retracted:** Oncotarget has completed its investigation of this paper. It was determined that the article contains multiple instances of image duplication and overlap, both internal to the paper (Figure 2 b-actin is overlapping with b-actin in Figure 4), and external with other published works. Three earlier submitted publications [1–3] share a significant number of duplicated and overlapping images with this paper, where figures of reference [1] were almost completely reproduced in the Oncotarget paper:

Figure 1, panels A and B are duplicates of Figure 2A, 2B [1] and panels 1C and 1D replicates Figure 3A, 3B [1], where data for the control is removed;
Figure 2 is a duplicate of Figure 4A [1] where data for the control is removed;
Figure 3 panels A, B and C are the duplicates of Figure 5 [1] panels B, A, and C, respectively; Figure 4 panels A and B duplicated panels A and B of Figure 6A and 6B and panel 4C duplicated Figure 7A [1].

Additionally, Figures 3B and 4B are duplicates of Figures 4F and 5F of [2] and Figure 4C b-actin is a duplicate of Figure 2B b-actin of [3] which also was found in Figure 6A of [4].

We also found that some images were duplicated in the later published papers, some of which are already retracted. Specifically, western blot for MMP-2 in Figure 1D was found as Bcl2 blot in Figure 7H of [5]; transwell assay images from Figures 3B and 4B were found in Figures 1B and 7D of [6], 3I, 5F and 6H of [7].

Authors were contacted and they informed us that the original data was lost. Taking into account all found inconsistencies, the Editorial decision has been made to retract this article. The authors were informed of this decision.

Original article: Oncotarget. 2017; 8:5936–5942. 5936-5942. https://doi.org/10.18632/oncotarget.13963
